# Differentiating effects of levodopa and subthalamic nucleus deep brain stimulation on motor features in Parkinson disease

**DOI:** 10.1016/j.prdoa.2025.100417

**Published:** 2025-12-16

**Authors:** Tiffanie A. Lee, Deepa Ramesh, Mwiza Ushe, Scott A. Norris, Samer D. Tabbal, Joel S. Perlmutter, John R. Younce

**Affiliations:** a University of North Carolina at Chapel Hill, Department of Neurology, 170 Manning Drive, Chapel Hill, NC 27599, USA; b Washington University in St Louis, Department of Neurology, 660 South Euclid Avenue, St Louis, MO 63110, USA; c Washington University in St Louis, Department of Radiology, St Louis, MO, USA; d Washington University in St Louis, Departments of Neuroscience, Physical Therapy, Occupational Therapy, St Louis, MO, USA

**Keywords:** Parkinson disease, Deep brain stimulation, Levodopa, Factor analysis

## Abstract

**Introduction:**

While deep brain stimulation of the subthalamic nucleus (STN DBS) is traditionally used to treat motor fluctuations in Parkinson disease (PD), recent progress in levodopa delivery systems may offer non-invasive alternatives to surgical options. To gain insight into symptoms more likely to be treated effectively by STN DBS or levodopa, we systematically compared the differential effects of levodopa and STN-DBS on specific parkinsonian motor components.

**Methods:**

We retrospectively analyzed 395 PD patients who received bilateral STN-DBS at Washington University (1999–2020). UPDRS-III was obtained in three conditions: preoperative OFF-medication, preoperative ON-medication, and postoperative ON-DBS/OFF-medication. Exploratory factor analysis identified a consensus structure across conditions. Treatment responses by factor were compared using Wilcoxon rank-sum tests and correlations were assessed with Kendall’s tau.

**Results:**

Both treatments significantly improved all motor features. STN-DBS showed slightly superior efficacy for upper body tremor (mean change: −3.84 vs −3.43, p < 0.001), while levodopa demonstrated much greater effectiveness for axial symptoms (−5.41 vs −2.65, p < 0.001), and slightly greater effectiveness for lower body tremor (−1.25 vs −1.06, p < 0.001), and lower body bradykinesia (−1.18 vs −0.91, p = 0.005). Correlations between treatment responses were weak but statistically significant for rigidity (τ = 0.158), bradykinesia (hand: τ = 0.118; leg: τ = 0.179), and axial symptoms (τ = 0.207).

**Conclusion:**

Levodopa and STN-DBS demonstrate similar but distinct therapeutic profiles across parkinsonian motor domains. However, weak correlations between treatment modalities for all motor features challenge the utility of levodopa responsiveness for DBS candidacy and support individualization of treatment selection based on symptom profile.

## Highlights

• Factor analysis identified six distinct parkinsonian motor domains across conditions.
• STN-DBS superior for upper body tremor; levodopa better for axial symptoms and legs.
• Weak correlations between levodopa and STN-DBS challenge use of levodopa response in candidacy.
• Treatment selection should target specific symptoms rather than global scores.

## 1 Introduction

Deep brain stimulation (DBS) of the subthalamic nucleus (STN) is an established adjunctive therapy for the treatment of motor symptoms of Parkinson disease (PD), often used when oral medications fail to adequately treat features such as motor fluctuations, tremor, and dyskinesia [1], [2]. Initially, response of motor symptoms to an oral levodopa challenge was considered the best predictor of response to STN DBS, with early studies demonstrating strong correlations between magnitude of DBS response and levodopa response [3], [4]. This relationship formed the foundation for patient selection criteria, with a sufficient response to oral levodopa challenge serving as a key determinant of STN DBS candidacy [2]. However, more recent studies have revealed weaker and more variable relationships between response to levodopa challenge and DBS effects, challenging the traditional assumption that the response to levodopa challenge predicts future effects of STN DBS [5], [6], [7], [8], [9]. Indeed, STN DBS is frequently employed for the treatment of tremor refractory to levodopa, while features such as freezing of gait and postural instability demonstrate complex and sometimes divergent relationships to both levodopa and STN DBS therapy [10], [11].

The clinical significance of understanding these differential responses has grown considerably in recent years due to advances in levodopa-based therapies. Improvements in the ability of levodopa formulations to manage motor fluctuations, including the development of continuous levodopa infusion (CLI) and extended-release (ER) formulations, have expanded the therapeutic options available to patients with advanced PD, including management of motor fluctuations, peak dose side effects, and OFF periods [12], [13]. These developments necessitate a more nuanced understanding of how specific parkinsonian features respond to each treatment modality to optimize patient selection between DBS and dopamine replacement strategies. The traditional approach of evaluating treatment response using global motor scores may obscure important differences in how individual motor components respond to different interventions, especially in light of substantial variability even within motor subtypes in PD [14], [15]. DBS may even exhibit differential effects on subcomponents within cardinal parkinsonian symptoms, such as bradykinesia [16]. As another example, while both tremor and gait are generally considered treatable by both DBS and levodopa, effects on each of these cardinal symptoms of PD likely differ between treatment modalities [11], [17]. The heterogeneity of PD motor symptoms and their varied responses to different treatments underscores the need for a systematic approach to understanding these relationships. While several studies have demonstrated differences in responses to STN DBS vs levodopa in specific symptoms and regional brain activity, a comprehensive analysis of differences in motor responses within a large dataset has not been performed to date [17], [18], [19]. To simplify the dataset for comparative analysis, we employed factor analysis, which provides a data-driven method for identifying latent variables underlying patterns of motor symptoms. This method can reveal hidden patterns among variables, including natural groupings of symptoms that may respond similarly to different therapeutic interventions [20].

The objective of this study was to determine how individual motor components of PD respond to treatment with oral levodopa compared with STN DBS, and to examine the relationship between response to oral levodopa challenge and STN DBS response at a granular level. To facilitate this comparison, we employed factor analysis to divide motor features of PD across multiple treatment conditions, including OFF-medication (OFF), ON-medication (ON), and OFF-medication/ON-DBS (DBS), attempting to create a consensus factor structure across conditions. This was followed by systematic comparison and correlation analysis between treatment modalities for symptoms within each consensus factor group. We hypothesized that levodopa and STN DBS would demonstrate similar overall effectiveness for most motor features except for tremor, which would be treated more effectively by STN DBS, and gait, which would be treated more effectively by levodopa. We also hypothesized that motor features would generally exhibit significant but modest correlations between levodopa challenge and STN DBS response, reflecting distinct but partially overlapping therapeutic mechanisms.

## 2 Methods

### 2.1 Overview

We conducted a retrospective analysis of all patients who received STN DBS for PD at Washington University in St. Louis (WUSTL) from 1999 to 2020. To compare the effectiveness of levodopa and STN DBS on specific components of parkinsonism, we used factor analysis to reduce the dimensionality of UPDRS-III scores obtained in three conditions: preoperative OFF-medication, preoperative ON-medication, and postoperative ON-stimulation/OFF-medication. We developed a consensus factor structure based on these analyses, then compared treatment responses for each factor group.

### 2.2 Participants

For this study, all patients who received STN DBS for PD at the WUSTL Movement Disorders Center from 1999 to 2020 (n = 618) were screened for retrospective analysis at the University of North Carolina at Chapel Hill (UNC). Inclusion criteria consisted of diagnosis of idiopathic PD and treatment with bilateral STN DBS and levodopa, as well as adequate preoperative and postoperative UPDRS-III data in all study conditions. No additional exclusionary criteria were used beyond those used to establish STN DBS candidacy at WUSTL, namely absence of structural brain abnormalities precluding safe electrode implantation, presence of dementia, history of encephalitis, stroke, and serious head injury. While patients with levodopa response under the traditional cutoff of 30 % are generally not considered good DBS candidates, levodopa response was considered in the context of the overall patient condition [2]. This resulted in 395 included subjects. Seventy-one (18 %) did not reach the 30 % levodopa response threshold but were nevertheless selected for STN DBS. The decision to pursue STN DBS was based on clinical judgement of the treating neurology and neurosurgical team, who considered STN DBS the best therapeutic option. Rationales for pursuing STN DBS in this cohort included levodopa-resistant tremor, borderline levodopa response, need to reduce levodopa dose, and suspected poor oral levodopa bioavailability due to absorption issues. This research was approved by the Institutional Review Boards at WUSTL (data acquisition and storage) and UNC (data analysis).

### 2.3 Surgical procedure

All patients underwent preoperative neuropsychological testing to confirm DBS candidacy and exclude dementia. Bilateral STN DBS was performed using contemporary DBS systems consisting of Medtronic electrodes connected to current-generation internal pulse generators (Activa SC, Kinetra SC, or Soletra SC) based on date of surgery. Electrode placement was verified using microelectrode recording, intraoperative electrode testing, and postoperative CT scans.

### 2.4 Clinical evaluation

Patients received standard of care DBS programming following electrode implantation. A conservative approach to DBS programming was employed, including monopolar review to identify electrodes with maximal therapeutic window, followed by gradual adjustments in amplitude, frequency, and pulse width over multiple programming visits. Patients were evaluated using the UPDRS-III at a single time point in three conditions: preoperative OFF-medication (≥12 h after last levodopa dose), preoperative ON-medication (withing 1 h following administration of optimized levodopa dose), and postoperative ON-stimulation/OFF-medication (approximately 12 months after electrode implantation with optimized stimulation parameters in OFF-medication condition). For preoperative conditions, we used UPDRS-III scores obtained during formal oral levodopa challenge testing closest to electrode implantation date within 12 months. For the postoperative condition, we used the scores closest to 12 months after electrode implantation. For patients without scores close to 12 months, we used the score closest to that timepoint, excluding scores from initial programming or more than 18 months after implantation. We also used these criteria to identify patients with postoperative OFF-DBS and OFF-medication scores to assess for effects of disease progression within the study timeframe. We calculated the change in scores for ON versus OFF conditions and DBS versus OFF conditions. UPDRS-III inter-rater reliability was validated for all UPDRS-III raters annually using video-based testing [21].

### 2.5 Statistical analysis

All analyses were performed using R version 4.4.2 [22].

Descriptive Statistics. We summarized demographic characteristics using frequencies for categorical variables and means with standard deviations for continuous variables. UPDRS-III scores were described for all conditions, and Kendall's tau correlations were computed between conditions for each item.

Factor Analysis. We performed exploratory factor analysis (EFA) on UPDRS-III data from all three conditions and their difference scores. Eigenvalues and scree plots guided selection of optimal factor count for each condition (OFF, ON, and DBS) and change between OFF and each treatment condition (i.e. response to levodopa and DBS). Each UPDRS-III item was assigned to the factor group with the highest item factor loading, representing the strength of relationship of that item with the underlying factor. Confirmatory factor analysis (CFA) validated the optimal factor structures using standard fit indices (CFI, RMSEA). RMSEA was used rather than CFI as the primary fit index due to high model complexity with >6 factors in all conditions [23], [24]. We compared factor structures across conditions and developed a consensus structure representing the most stable symptom groupings.

Treatment Comparisons. For each consensus factor group, we calculated mean changes (absolute and relative) from baseline for levodopa (ON vs OFF) and DBS (DBS vs OFF) conditions and averaged within factor group to create a composite score. We used Wilcoxon rank-sum tests to compare treatment effects between modalities by factor group. We also computed conditional probabilities of STN DBS improvement given levodopa improvement for each factor using 2 methods: 1) probability of ≥ 1 point improvement in STN DBS improvement given ≥ 1 point improvement in levodopa improvement, and 2) probability of DBS improvement ≥ levodopa improvement. We also computed correlations (Kendall’s tau) between relative response in each factor for each treatment modality, excluding patients with a score of zero in the baseline “OFF” condition for that factor. For significance testing, we corrected for multiple comparisons using the Benjamini-Hochberg False Discovery Rate (FDR).

## 3 Results

Patient demographic data and clinical characteristics are summarized in Table 1. Patients had significant reduction in UPDRS-III from both levodopa and STN DBS (Table 1), with greater overall response to levodopa than STN DBS (mean change −19.1 versus −15.8, p < 0.001). To account for any effect of disease progression between preoperative levodopa and postoperative STN DBS scores, we identified a subgroup (n = 58) with postoperative OFF-DBS and OFF-levodopa scores within the same timeframe as used for postoperative STN DBS scores. Within this subgroup, preoperative OFF (mean 44.4, SD 14.6) and postoperative OFF/OFF (42.4, SD 14.7) were similar, suggesting that disease progression was minimal within the study timeframe and not primarily responsible for observed differences between levodopa and STN DBS effects.

**Table 1:** Demographics and clinical data.

Sex	239 male, 156 female
Handedness	353 right, 42 left
Years of education	15.0 (4.3) years
Age of PD symptom onset	51.2 (10.1) years
Age at DBS	64.3 (8.9) years
Years since diagnosis	13.0 (6.5) years
UPDRS-III baseline (OFF-levodopa)	40.9 (11.3)
UPDRS-III change from levodopa (absolute / percent)	−19.1 (10.0) / −46.1 (18.9) %
UPDRS-III change from DBS (absolute / percent)	−15.8 (11.7) / −37.0 (25.6) %

Values reported as mean (standard deviation).

Factor analysis revealed generally consistent patterns across conditions, with optimal factor structures summarized in Table 2. The consensus factor structure identified six primary parkinsonian factors: rigidity, upper body bradykinesia, lower body bradykinesia, upper extremity tremor (rest and action combined), lower extremity rest tremor, and axial symptoms (Table 2). Comparative analysis of levodopa and STN DBS effects among all patients revealed significant differences across multiple factors (Table 3a). STN DBS had a greater effect than levodopa on upper body tremor (DBS: −3.84 vs LD: –3.43, p < 0.001), while levodopa had a greater effect than STN DBS on axial symptoms (LD: −5.41 vs DBS −2.65, p < 0.001), lower body tremor (LD: −1.25 vs DBS: −1.06, p < 0.001), and lower body bradykinesia (LD: −1.18 vs DBS: −0.91, p = 0.005). Rigidity and upper body bradykinesia responses did not differ significantly between treatment modalities. When the canonically DBS-nonresponsive symptoms of speech and facial expression were excluded from the axial domain, we observed similar relative responses to LD and DBS compared with the complete axial domain (LD response of 45 % vs 49 % and DBS response of 22 % vs 25 %, respectively). For all factors, both STN DBS and levodopa significantly reduced scores compared with the OFF condition. Weak but statistically significant correlations between STN DBS and response to levodopa challenge were observed for rigidity, bradykinesia of hand and leg, and axial symptoms (Table 3a). Notably, STN DBS and levodopa response to tremor in upper and lower body did not correlate between treatment modalities.

**Table 2:** Optimal factor structures of UPDRS-III by treatment condition and consensus factor structure. Each item was assigned to the factor group with the highest factor loading (i.e. strongest relationship between item and factor group). RMSEA < 0.08 indicates acceptable fit.

	OFF	ON	DBS	ON vs OFF	DBS vs OFF	Consensus
	RMSEA = 0.066	RMSEA = 0.055	RMSEA = 0.061	RMSEA = 0.051	RMSEA = 0.054	
Rigidity Neck	1	1	1	1	1	1
Rigidity RUE	1	1	1	1	1	1
Rigidity LUE	1	1	1	1	1	1
Rigidity RLE	1	1	1	1	1	1
Rigidity LLE	1	1	1	1	1	1
Finger Taps RUE	2	2	2	2	2	2
Hand Movement RUE	2	2	2	2	2	2
Rapid Alternating RUE	2	2	2	2	2	2
Finger Taps LUE	3	2	3	2	2	2
Hand Movement LUE	3	2	3	2	2	2
Rapid Alternating LUE	3	2	3	2	2	2
Leg Agility RLE	2	3	4	2	3	3
Leg Agility LLE	3	3	4	2	3	3
Action Tremor RUE	4	4	5	3	4	4
Action Tremor LUE	4	4	5	3	4	4
Rest Tremor RUE	4	4	5	4	4	4
Rest Tremor LUE	4	4	5	3	4	4
Rest Tremor Face	4	6	7	4	4	4
Rest Tremor RLE	5	5	6	4	5	5
Rest Tremor LLE	5	5	6	4	5	5
Speech	6	7	7	5	6	6
Expression	6	7	7	5	6	6
Posture	6	7	8	5	6	6
Body Bradykinesia	6	7	8	6	6	6
Gait	6	7	8	6	6	6
Arising	6	7	8	6	6	6
Postural Stability	6	7	8	6	6	6

**Table 3a:** Response to levodopa vs DBS among all patients, organized by consensus factor structure, expressed as mean (SD).

	Levodopa response	DBS response	tau
Rigidity	−3.65 (3.15)	−3.48 (3.23)	0.158†
Bradykinesia (Hand)	−4.29 (3.26)	−3.85 (4.16)	0.118†
Bradykinesia (Leg)*	−1.18 (1.47)	−0.91 (1.65)	0.179†
Tremor (Upper Body)*	−3.43 (3.26)	−3.84 (3.47)	0.073
Tremor (Lower Body)*	−1.25 (1.79)	−1.06 (1.73)	−0.076
Axial*	−5.41 (3.44)	−2.65 (4.31)	0.207†

Tau indicates Kendall rank correlation coefficient between conditions.

* Indicates significant difference between treatment conditions at p < 0.05, FDR-corrected.

† Indicates significant correlation between treatment conditions at p < 0.05, FDR-corrected.

To highlight the DBS responsiveness of patients often not considered DBS candidates due to weak responses to oral levodopa challenge, we performed a subgroup analysis in patients with a < 30 % response to levodopa challenge, which included 71 of the 395 subjects. Of these patients, 40 had medication-resistant tremor and 21 had a borderline levodopa response (between 20 % and 30 %). A subgroup analysis revealed that patients with low response to levodopa challenge had generally superior motor responses to DBS than to levodopa across all symptom domains, excepting lower extremity tremor (Table 3b).

**Table 3b:** Response to levodopa vs DBS among patients with < 30 % LD response, organized by consensus factor structure, expressed as mean (SD).

	Levodopa response	DBS response	p
Rigidity*	−1.90 (2.62)	−3.17 (3.75)	0.0027
Bradykinesia (Hand)*	−1.43 (2.16)	−3.60 (−3.50)	0.0096
Bradykinesia (Leg)*	−0.25 (1.17)	−0.73 (1.74)	0.02868
Tremor (Upper Body)*	−1.46 (1.70)	−2.45 (3.07)	0.00202
Tremor (Lower Body)	–0.30 (0.91)	−0.42 (0.96)	0.251
Axial*	−2.33 (2.40)	0.58 (4.10)	0.00202

* Indicates significant difference between treatment conditions at p < 0.05. P values were corrected for multiple comparisons using FDR.

**Fig. 1:**
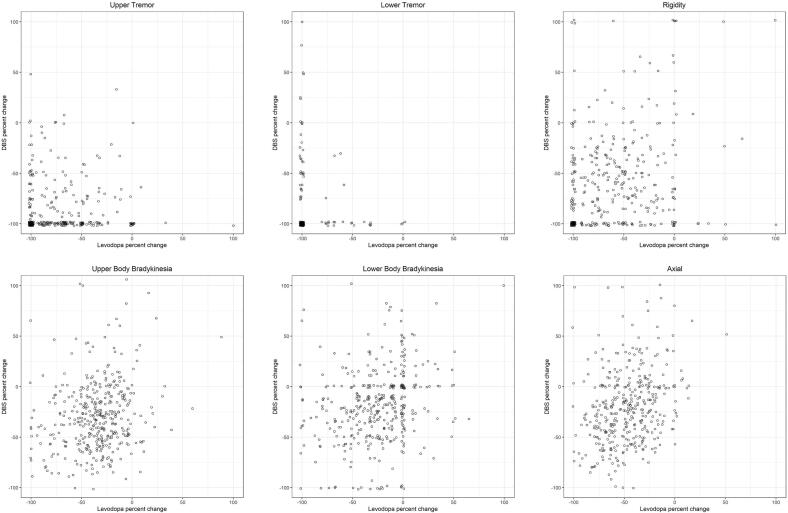
Relative response to levodopa and DBS for each parkinsonian factor composite score in percent change. Instances with baseline OFF score of zero were excluded. To facilitate visualization of overall trends, jitter of up to 2 percent was applied and outliers of over 100% change were not shown. Cluster at −100/-100 included 146 of 349 subjects for upper tremor, 112 of 173 subjects for lower tremor, and 55/376 subjects for rigidity.

We plotted the relationships between relative change in parkinsonian factor composite score after STN DBS versus after levodopa (Fig. 1) and calculated probability of STN DBS response given response to levodopa challenge within a particular factor. We computed percent chance of DBS response of *at least 1 point* given levodopa response of at least 1 point (Supplementary Table 1a) and percent chance of STN DBS response of *at least as great* as levodopa response (Supplementary Table 1b). STN DBS response rates for most factors were high given response to levodopa challenge (over 80 %), with exceptions in lower body bradykinesia (61 %) and axial symptoms (70 %). STN DBS response rates of *at least as great* as response to levodopa challenge were more mixed, with upper body tremor (82 %) and lower body tremor (87 %) responding most consistently, while axial symptoms responded the least consistently (29 %).

## 4 Discussion

This study presents a comprehensive analysis of the differential effects of levodopa and STN DBS on specific motor components of Parkinson's disease, utilizing factor analysis to identify distinct symptom clusters. Our results demonstrate that while both levodopa and STN DBS provide significant benefit for most parkinsonian motor features, their therapeutic profiles differ across specific symptom domains. Most notably, STN DBS showed slightly greater efficacy for upper body tremor, while levodopa demonstrated greater effectiveness for axial symptoms, and slightly greater effectiveness for lower body tremor and lower body bradykinesia. Correlations between response to levodopa challenge and STN DBS response were weak for all parkinsonian symptoms, and variability in response was high for both treatment modalities. The poor relationship between levodopa and STN DBS responses is further supported by the subgroup of patients with substandard responses to levodopa challenge, where STN DBS had a greater efficacy in nearly all symptom domains among those with a weak levodopa response. Both STN DBS and levodopa significantly improved all motor features compared with the OFF-medication condition. The effect size between treatment conditions was generally small for all symptom groups, except for axial features, which showed a 2.76 point greater improvement in response to LD than to DBS from the OFF condition. While most patients experienced at least a 1 point improvement in response to STN DBS and LD for all factors, most patients experienced greater improvement to upper body and lower body tremor from DBS than LD, and a greater improvement to axial symptoms from LD than DBS.

Our factor analysis revealed a robust factor structure that varied only slightly across treatment conditions, providing validation for the natural clustering of parkinsonian symptoms within a six-factor consensus structure. While these factors generally reflected agreement with cardinal features of Parkinson disease, subdivision by anatomical region was also present in some symptoms. Interestingly, rigidity appeared to consistently operate as a global unit, while bradykinesia and tremor were divided anatomically in most treatment conditions. Bradykinesia exhibited the most anatomical variability, with upper/lower body splits in some treatment conditions, and left/right splits in others. Consistent grouping of symptoms involving gait and posture with features such as speech and facial expression was surprising, as was the grouping of action tremor with rest tremor in all but 1 condition. These observations support the value of using data-driven methods to reduce dimensionality in motor parkinsonism rather than assuming the validity of grouping symptoms *a priori*.

The superior efficacy of STN DBS for upper body tremor aligns with established clinical experience and supports the use of DBS in patients with medically refractory tremor [11]. The effect size difference, while statistically significant, was relatively modest (DBS: −3.84 vs levodopa: −3.43), suggesting that both treatments provide substantial tremor control. However, the high conditional probability of DBS tremor response indicates that patients who respond well to levodopa for tremor are very likely to also experience benefit from DBS. Interestingly, this did not hold true for lower body tremor, which responded slightly better to levodopa than DBS, supporting prior reports that leg tremor exhibits weaker responses to STN DBS than arm tremor [25]. In both cases, the correlation between tremor response to levodopa challenge and STN DBS was extremely weak, supporting the notion of using STN DBS as a therapeutic option even when tremor is not responsive to levodopa. The observed low correlations in domains even where conditional probabilities were high suggests that while levodopa and DBS often affect the same symptom, the *magnitude* of this effect is generally unrelated.

Conversely, the superior efficacy of levodopa for axial symptoms presents important clinical considerations. Axial symptoms, including speech, hypomimia, camptocormia, gait abnormality, and postural stability, are particularly disabling features of advanced PD that significantly impact quality of life. While freezing of gait (FOG) was not specifically tested in this study due to use of the traditional UPDRS-III, FOG has been correlated with other axial symptoms, including festination and postural instability [26], [27]. The overall poor effect of STN DBS compared with levodopa in the axial domain, including many cases of worsening axial symptoms following STN DBS, provide further support for optimization of dopaminergic therapy as a priority, particularly with the availability of advanced levodopa formulations such as continuous levodopa infusion and extended-release preparations [12], [13]. Even in those receiving DBS, this distinction in effect may warrant caution with aggressive STN DBS programming and levodopa reduction in patients with axial symptoms, even when well-controlled on current medication regimen prior to DBS. Furthermore, while this study only included STN DBS patients, other studies have reported similar effects on axial symptoms between STN DBS and DBS of the internal globus pallidus (GPI) [28]. The effects of STN DBS on gait, akinesia, and freezing are complex, and both substantial improvement and paradoxical exacerbation of these symptoms have been reported, although current ability to predict this outcome pre-operatively remains lacking [29], [30], [31]. While the grouping of facial expression and speech with gait and postural stability was unexpected, it is unclear whether this represents a shared physiological substrate or simply represents a grouping of symptoms with a similarly less robust response to STN DBS. The grouping of body bradykinesia and hypokinesia with axial symptoms such as posture and facial expression rather than appendicular bradykinesia further suggests that the designation of this item as “bradykinesia” may be inaccurate, which may better be described as an independent phenomenon as “akinesia” or “hypokinesia”, an observation supported by a change in terminology in the updated MDS-UPDRS [20], [32].

The weak correlations between response to levodopa challenge and STN DBS responses across most motor domains support the distinct mechanisms of action of these therapies. While strength of correlation is limited by variability in testing, we mitigated this here by using annually validated raters in a single center. This finding has important implications for patient selection in DBS, as it suggests that oral levodopa response testing as commonly used in isolation may poorly reflect eventual DBS outcomes, particularly for specific symptom domains. These low correlations also align with a growing body of evidence showing the limited utility of levodopa challenge as a useful tool for evaluating DBS candidacy [5], [7]. Furthermore, these findings are consistent with emerging evidence that DBS effects involve complex interactions with basal ganglia circuitry that may alter pathological oscillations and affect networks affected by PD in a distinct fashion from dopamine replacement [33]. Classically, DBS has been thought to act primarily on the indirect pathway as compared with the less specific dopaminergic action provided by levodopa, although this likely does not account for the entire mechanism of STN DBS [34]. However, this more selective mechanism of STN DBS within the basal ganglia may allow for neuroanatomical specificity in DBS programming, dependent in part on exact placement of electrodes within subunits of the STN, and may have advantages for action on specific tracts implicated in parkinsonian tremor [35], [36]. This concept of neuroanatomical selectivity may also explain the differences in factor structure in the levodopa vs the DBS state, with bradykinesia responses to STN DBS more divided by laterality than responses to levodopa (Table 2). Along these lines, the tendency for rigidity to operate as a global unit, as opposed to the anatomical divisions in tremor and bradykinesia, may indicate that the transcortical pathway involved in generation of long-latency reflexes implicated in rigidity exhibit less neuroanatomical specificity than those in other cardinal parkinsonian features [37], [38]. From another perspective, this greater neuroanatomical selectivity in DBS compared with levodopa may also influence which symptoms are targeted by clinicians. While tremor responds rapidly to STN DBS, bradykinesia is considerably slower to respond, while axial symptoms may not fully respond for several hours [39]. This rapidity of response may lead clinicians to “anchor” to tremor response and rigidity over axial and gait symptoms in their programming strategy, and may plausibly account for some of the difference in STN DBS response for these symptoms. Future studies may include use of programming strategies using electrophysiological responses as a biomarker rather than anchoring on clinical response, potentially reducing this bias.

Our findings suggest a framework for clinical decision-making that considers specific symptom profiles rather than global motor scores. Patients with predominantly tremor-dominant disease, particularly those with upper body tremor, may be excellent candidates for STN DBS even if their overall response to levodopa challenge is modest. Conversely, patients with prominent axial symptoms may benefit more from optimization of dopaminergic therapy, including consideration of advanced levodopa formulations, before proceeding to surgical intervention. Furthermore, even patients with globally weak response to levodopa challenge are likely to experience a substantial improvement from STN DBS, with greater average improvement from DBS than from levodopa across nearly all symptom domains. The weak correlations between levodopa challenge and DBS response, as well as the modest conditional probability of STN DBS response being at least as great as levodopa response, suggest that response to levodopa challenge should be interpreted cautiously in the context of STN DBS evaluation. These data suggest that use of a weak response to levodopa challenge to exclude patients from STN DBS candidacy is flawed, and treatment of response to levodopa challenge >30 % as an essential criterion for DBS candidacy needlessly prevents many patients from receiving an effective treatment for advancing motor symptoms of PD. It should be noted that this does not imply that patients with levodopa non-responsive disease (i.e. per MDS clinical diagnostic criteria for Parkinson disease) should receive STN DBS, only that the *magnitude* of response to oral levodopa challenge as measured by UPDRS-III is likely a poor tool for evaluating potential STN DBS effects [40].

Several limitations should be acknowledged in interpreting these results. First, the retrospective design limits our ability to control for potential confounding factors such as medication timing, exact electrode placement and consequent contact selection, stimulation parameters, and disease progression, although in our validation cohort we did not observe any global disease progression within the study timeframe. Second, our cohort was predominantly white (92 %), potentially limiting generalizability to minoritized populations. Third, the UPDRS-III, while widely used and validated, may not capture all relevant aspects of motor function, particularly subtle changes in movement quality or patient-reported outcomes. Furthermore, while the use of the traditional UPDRS-III allowed for a large sample size dating to well before the updated MDS-UPDRS-III, it does lack items that assess lower body bradykinesia and freezing of gait in more detail, thus limiting sensitivity of this analysis in these motor domains. Additionally, levodopa response was used in clinical patient selection for STN DBS thus potentially reducing generalizability of findings to those without robust levodopa response. However, a substantial subgroup of patients within this cohort included in the analysis (71 out of 395 total) had a levodopa response below the traditional cutoff of 30 %, supporting the generalizability of these findings. While the single-center nature of this study allowed for high consistency in data collection (including using raters validated on an annual basis), surgical methodology, and clinical decision-making, it also may limit generalizability to centers where different approaches to these questions are used. In particular, this center generally uses a conservative approach to DBS programming and focuses on maximizing medical therapy prior to DBS, while some centers promote a more DBS-centric approach earlier in the progression of PD [41], [42]. Finally, the retrospective nature of our study represents a limitation. Although a prospective study would be valuable to validate the findings in this study, a strength of our retrospective dataset was enabling the large single-center sample size employed here. Future prospective studies may have the advantage of including pallidal DBS in addition to STN DBS, inclusion of additional motor data such as repeated UPDRS examinations and kinematic data, as well as potentially being able to examine electrode localization and electrophysiological data.

## 5 Conclusions

This study provides quantitative evidence for differential therapeutic profiles of levodopa and STN DBS across specific parkinsonian motor domains. These findings support a personalized approach to treatment selection that considers individual symptom profiles and challenge the traditional reliance on global response to levodopa challenge as a major criterion for DBS candidacy in PD. As advanced therapies for PD continue to evolve, understanding these differential treatment effects will be crucial for optimizing treatment selection on an individual basis in order to maximize therapeutic benefit.

## Funding Sources

This study was supported by the American Parkinson Disease Association (APDA) Advanced Research Center at Washington University, the Missouri Chapter of the APDA, the Barnes-Jewish Hospital Foundation, and the National Institutes of Health (K23 NS121630, R01 NS041509).

## CRediT authorship contribution statement

**Tiffanie A. Lee:** Writing – review & editing, Writing – original draft, Visualization, Methodology, Investigation, Formal analysis. **Deepa Ramesh:** Writing – review & editing, Writing – original draft, Investigation, Data curation. **Mwiza Ushe:** Writing – review & editing, Investigation. **Scott A. Norris:** Writing – review & editing, Investigation. **Samer D. Tabbal:** Writing – review & editing, Investigation. **Joel S. Perlmutter:** Writing – review & editing, Supervision, Software, Resources, Investigation, Funding acquisition, Conceptualization. **John R. Younce:** Conceptualization, Methodology, Formal analysis, Investigation, Data curation, Resources, Writing – original draft, Writing – review & editing, Visualization, Supervision, Project administration, Funding acquisition.

## Declaration of competing interest

The authors declare the following financial interests/personal relationships which may be considered as potential competing interests: John Younce reports financial support was provided by National Institutes of Health. Joel Perlmutter reports financial support was provided by National Institutes of Health. Joel Perlmutter reports financial support was provided by American Parkinson Disease Association. Joel Perlmutter reports financial support was provided by Saint Louis American Parkinson Disease Association. Joel Perlmutter reports financial support was provided by Foundation for Barnes-Jewish Hospital. If there are other authors, they declare that they have no known competing financial interests or personal relationships that could have appeared to influence the work reported in this paper.
