# Supplementary material for: Differentiating effects of levodopa and subthalamic nucleus deep brain stimulation on motor features in Parkinson disease
Source: Clin Park Relat Disord. 2025 Dec 16;14:100417. doi: 10.1016/j.prdoa.2025.100417 (PMC12775982; doi:10.1016/j.prdoa.2025.100417)
Supplement: Supplementary Data 1 [file mmc1.docx]

Supplementary Table 1: Conditional probabilities of DBS response given levodopa response, organized by consensus factor structure. A: Probabilities of DBS response of *at least* 1 point given levodopa response of *at least* 1 point. B: Probabilities of DBS response of *at least as great* as levodopa response.

A

| Rigidity | 0.8798799 |
| --- | --- |
| Brady_Hand | 0.8091168 |
| Brady_Leg | 0.6106195 |
| Upper_Tremor | 0.961039 |
| Lower_Tremor | 0.9285714 |
| Axial | 0.701897 |

B

| Rigidity | 0.5964912 |
| --- | --- |
| Brady_Hand | 0.4759494 |
| Brady_Leg | 0.4987342 |
| Upper_Tremor | 0.8202532 |
| Lower_Tremor | 0.8708861 |
| Axial | 0.2911392 |
