# Supplementary material for: Differentiating effects of levodopa and subthalamic nucleus deep brain stimulation on motor features in Parkinson disease
Source: Clin Park Relat Disord. 2025 Dec 16;14:100417. doi: 10.1016/j.prdoa.2025.100417 (PMC12775982; doi:10.1016/j.prdoa.2025.100417)
Supplement: Supplementary Data 2 [file mmc2.docx]

Supplementary Table 2: Optimal factor structures of UPDRS-III by treatment condition and consensus factor structure, with item-wise factor loadings included.

|  | OFF | ON | DBS | ON vs OFF | DBS vs OFF | Consensus |
| --- | --- | --- | --- | --- | --- | --- |
|  | RMSEA=0.066 | RMSEA=0.055 | RMSEA=0.061 | RMSEA=0.051 | RMSEA=0.054 |  |
| Rigidity Neck | Factor 1 (0.6) | 1 (0.6) | 1 (0.6) | 1 (0.4) | 1 (0.4) | 1 |
| Rigidity RUE | 1 (0.7) | 1 (0.5) | 1 (0.7) | 1 (0.6) | 1 (0.6) | 1 |
| Rigidity LUE | 1 (0.7) | 1 (0.6) | 1 (0.7) | 1 (0.7) | 1 (0.6) | 1 |
| Rigidity RLE | 1 (0.8) | 1 (0.7) | 1 (0.7) | 1 (0.7) | 1 (0.7) | 1 |
| Rigidity LLE | 1 (0.7) | 1 (0.8) | 1 (0.8) | 1 (0.7) | 1 (0.7) | 1 |
| Finger Taps RUE | Factor 2 (0.6) | 2 (0.5) | 2 (0.5) | 2 (0.4) | 2 (0.6) | 2 |
| Hand Movement RUE | 2 (0.6) | 2 (0.7) | 2 (0.7) | 2 (0.5) | 2 (0.7) | 2 |
| Rapid Alternating RUE | 2 (0.6) | 2 (0.6) | 2 (0.4) | 2 (0.6) | 2 (0.5) | 2 |
| Finger Taps LUE | Factor 3 (0.6) | 2 (0.5) | 3 (0.7) | 2 (0.4) | 2 (0.5) | 2 |
| Hand Movement LUE | 3 (0.7) | 2 (0.7) | 3 (0.5) | 2 (0.4) | 2 (0.5) | 2 |
| Rapid Alternating LUE | 3 (0.6) | 2 (0.6) | 3 (0.6) | 2 (0.5) | 2 (0.4) | 2 |
| Leg Agility RLE | 2 (0.6) | 3 (1) | 4 (0.9) | 2 (0.4) | 3 (0.6) | 3 |
| Leg Agility LLE | 3 (0.4) | 3 (0.5) | 4 (0.6) | 2 (0.4) | 3 (0.7) | 3 |
| Action Tremor RUE | Factor 4 (0.8) | 4 (0.7) | 5 (0.8) | 3 (0.6) | 4 (0.7) | 4 |
| Action Tremor LUE | 4 (0.8) | 4 (0.8) | 5 (0.8) | 3 (0.7) | 4 (0.7) | 4 |
| Rest Tremor RUE | 4 (0.5) | 4 (0.3) | 5 (0.4) | 4 (0.4) | 4 (0.4) | 4 |
| Rest Tremor LUE | 4 (0.5) | 4 (0.4) | 5 (0.5) | 3 (0.4) | 4 (0.4) | 4 |
| Rest Tremor Face | 4 (0.2) | 6 (0.3) | 7 (0.1) | 4 (0.2) | 4 (0.2) | 4 |
| Rest Tremor RLE | Factor 5 (0.8) | 5 (0.6) | 6 (0.7) | 4 (0.8) | 5 (0.8) | 5 |
| Rest Tremor LLE | 5 (0.8) | 5 (0.6) | 6 (0.8) | 4 (0.6) | 5 (0.7) | 5 |
| Speech | Factor 6 (0.4) | 7 (0.4) | 7 (0.6) | 5 (0.3) | 6 (0.3) | 6 |
| Expression | 6 (0.5) | 7 (0.6) | 7 (0.8) | 5 (0.6) | 6 (0.5) | 6 |
| Posture | 6 (0.6) | 7 (0.6) | 8 (0.5) | 5 (0.4) | 6 (0.4) | 6 |
| Body Bradykinesia | 6 (0.6) | 7 (0.6) | 8 (0.5) | 6 (0.3) | 6 (0.6) | 6 |
| Gait | 6 (0.7) | 7 (0.7) | 8 (0.8) | 6 (0.5) | 6 (0.6) | 6 |
| Arising | 6 (0.7) | 7 (0.7) | 8 (0.8) | 6 (0.5) | 6 (0.5) | 6 |
| Postural Stability | 6 (0.7) | 7 (0.5) | 8 (0.7) | 6 (0.4) | 6 (0.6) | 6 |
